# Uncertainty Quantification of Methods Used to Measure Methane Emissions of 1 g CH_4_ h^−1^

**DOI:** 10.3390/s23229246

**Published:** 2023-11-17

**Authors:** Stuart N. Riddick, Mercy Mbua, John C. Riddick, Cade Houlihan, Anna L. Hodshire, Daniel J. Zimmerle

**Affiliations:** 1 Energy Institute, Colorado State University, Fort Collins, CO 80524, USA; mercy.mbua@colostate.edu (M.M.); anna.hodshire@colostate.edu (A.L.H.); dan.zimmerle@colostate.edu (D.J.Z.); 2 Independent Researcher, Lockerbie DG11 2DU, Scotland, UK

**Keywords:** methane, abandoned wells, quantification, uncertainty

## Abstract

The recent interest in measuring methane (CH_4_) emissions from abandoned oil and gas wells has resulted in five methods being typically used. In line with the US Federal Orphaned Wells Program’s (FOWP) guidelines and the American Carbon Registry’s (ACR) protocols, quantification methods must be able to measure minimum emissions of 1 g of CH_4_ h^−1^ to within ±20%. To investigate if the methods meet the required standard, dynamic chambers, a Hi-Flow (HF) sampler, and a Gaussian plume (GP)-based approach were all used to quantify a controlled emission (*Q_av_*; g h^−1^) of 1 g of CH_4_ h^−1^. After triplicate experiments, the average accuracy (*A_r_*; %) and the upper (*U_u_*; %) and lower (*U_l_*; %) uncertainty bounds of all methods were calculated. Two dynamic chambers were used, one following the ACR guidelines, and a second “mobile” chamber made from lightweight materials that could be constructed around a source of emission on a well head. The average emission calculated from the measurements made using the dynamic chamber (*Q_av_* = 1.01 g CH_4_ h^−1^, *A_r_* = +0.9%), the mobile chamber (*Q_av_* = 0.99 g CH_4_ h^−1^, *A_r_* = −1.4%), the GP approach (*Q_av_* = 0.97 g CH_4_ h^−1^, *A_r_* = −2.6%), and the HF sampler (*Q_av_* = 1.02 g CH_4_ h^−1^, *A_r_* = +2.2%) were all within ±3% of 1 g of CH_4_ h^−1^ and met the requirements of the FOWP and ACR protocols. The results also suggest that the individual measurements made using the dynamic chamber can quantify emissions of 1 g of CH_4_ h^−1^ to within ±6% irrespective of the design (material, number of parts, geometrical shape, and hose length), and changes to the construction or material specifications as defined via ACR make no discernible difference to the quantification uncertainty. Our tests show that a collapsible chamber can be easily constructed around the emission source on an abandoned well and be used to quantify emissions from abandoned wells in remote areas. To our knowledge, this is the first time that methods for measuring the CH_4_ emissions of 1 g of CH_4_ h^−1^ have been quantitively assessed against a known reference source and against each other.

## 1. Introduction

Methane (CH_4_) gas is a key driver in climate change and the main constituent of natural gas (NG), which has been extracted from the subsurface in the US since the 1860s and used as a fuel [1]. Historically, once a well ceases to produce economically viable oil or NG, the above-ground infrastructure is removed, and any CH_4_ that is left in the reservoir continues to be emitted to the atmosphere. Only in recent years have non-producing wells been plugged with concrete, capped, sealed, and buried under six feet of soil. It is currently estimated that there are over 2 million unplugged and abandoned wells in the US alone [2], with the state average emissions from plugged wells ranging from 0.3 g CH_4_ h^−1^ in California to 12 g CH_4_ h^−1^ in Pennsylvania [3], and the state average emissions from unplugged wells ranging from 3 g CH_4_ h^−1^ in West Virginia to 534 g CH_4_ h^−1^ in Colorado [4].

As part of the 2021 Bipartisan Infrastructure Law (BIL; Public Law 117-58), the Federal Orphaned Wells Program’s (FOWP) guidelines [5] were published to describe how the federal program’s reporting requirements for CH_4_ emissions reductions are to be met, as described in Section 40601 (orphaned well site plugging, remediation, and restoration) of Title V (Methane Reduction Infrastructure). The guidelines state that methods used to quantify emissions should have a minimum quantification limit of 1 g CH_4_ h^−1^ and a quantification uncertainty of ±30%. The guidelines recommend that EPA Method 21 be followed [6] to estimate emissions when a well head or other infrastructure is present. Method 21 does not specify which instrument/detector should be used, but it states that it must be an intrinsically safe, pump-driven, non-CH_4_-specific volatile organic compound (VOC) monitoring instrument with a scale that is readable to ±2.5% of the specified leak definition concentration [6]. Method 21 is not used to quantify emissions from individual sources only to determine if the VOCs are leaking. The FOWP guidelines also suggest the use of other quantification methods including high volume samplers, static and dynamic chambers, and combinations of various (measurement/sampling) techniques. No specific details are given of these methods in the guidelines.

The American Carbon Registry (ACR) published a methodology to provide a quantification framework for the creation of carbon offset credits through CH_4_ emission reduction resulting from plugging abandoned oil and gas wells [7]. The ACR prescribes the use of a CH_4_-specific gas detector and a static or dynamic chamber. No minimum quantification limit is set in the ACR guidelines, but quantification uncertainty is defined at ±20%, and firm specifications are presented for the chamber’s design. These specifications are that the chamber footprint should cover the well and up to a one-meter buffer around the well and be constructed from a material that has no degassing or sorption of CH_4_. The chamber should have a detachable base, inserted between 2 and 6 cm into the ground surface, and be installed before any measurement. The upper chamber component should have a vent tube with the length being dependent on the wind speed. The chamber must be airtight. Fans must be used to ensure that the CH_4_ inside the chamber is well mixed and positioned to provide sufficient circulation without affecting the pressures. The analyzer must be able to analyze and provide CH_4_-specific concentrations from 1 ppmv to 100% CH_4_ with a measurement frequency of 1 Hz and a precision of 1 ppmv. From the ACR, it is unclear if the chamber should be sized to include well heads or other infrastructure if they are present.

Typical quantification methods that have been used to measure CH_4_ from abandoned oil and gas wells extend beyond the chamber method recommended by the ACR methodology and include those that touch/enclose the well head and downwind measurements. Downwind approaches are typically used when the well head, Christmas tree, or pumpjack is too big to fit in a chamber or too awkward to be enclosed by a Hi-Flow (HF) sampler bag. Examples of direct methods are static chambers [8], dynamic flux chambers [4,9,10], and Hi-Flow (HF) sampling [11,12,13]. Downwind methods include Gaussian-based plume (GP) approaches [14,15] and Lagrangian dispersion models [16,17]. Other methods can be used to quantify emissions but are not well suited to measure the relatively small amounts of CH_4_ that are typically emitted from abandoned wells [3,4,11,18]. For example, the quantification limit of optical gas imaging (OGI) cameras is higher than the average abandoned well’s emission by at least a factor of two [19,20], tracer release requires road access, which is problematic for most abandoned wells as they are generally in wooded areas [21], aircraft survey methods have a lower quantification limit of 1 kg of CH_4_ h^−1^ [22], and satellites have a lower quantification limit of 100 kg of CH_4_ h^−1^ [23]. To date, the Hi-Flow sampler is the only ACR-approved non-chamber method.

An evaluation of the methods that are typically used to quantify CH_4_ emissions from abandoned oil and gas wells is presented in [24]. The average accuracy rates of the three experiments (*A_r_*; %) were presented for static chambers, dynamic chambers, HF measurements, GP modeling and Lagrangian stochastic (Ls) methods, and they were calculated from controlled CH_4_ release experiments between 40 and 200 g of CH_4_ h^−1^. The static chamber data were not presented in the study by [24] because of safety concerns during the experiments. Both the dynamic chamber (*A_r_* = −10%, −8%, and −10% at emission rates of 40, 100, and 200 g CH_4_ h^−1^, respectively) and HF (*A_r_* = −18%, −16%, and −18%) repeatedly underestimated the emission, but the dynamic chamber had better accuracy. The standard deviations of the emissions from these direct measurement methods remained relatively constant for emissions between 40 and 200 g CH_4_ h^−1^. The downwind GP method significantly overestimated the emissions (*A_r_* = +86%, +57%, +29%) [24],; however, it was noted that both the accuracy and precision were likely to improve if the measurements were taken directly downwind. 

From these experiments, it can be seen that the dynamic chambers and HF meet the uncertainty thresholds of both the FOWP guidelines (±30%) and the ACR criteria (±20%) for emissions between 40 and 200 g CH_4_ h^−1^, but it is unclear how these methods perform while measuring emissions of 1 g of CH_4_ h^−1^, i.e., the minimum quantification limit for the FOWP. To address this, we (1) determine the uncertainty in the quantification of the dynamic chamber, HF sampler, and GP approach for an emission rate of 1 g of CH_4_ h^−1^; (2) describe the construction and test the uncertainty of a novel “mobile” dynamic chamber; and (3) provide recommendations for CH_4_ quantification in abandoned wells.

## 2. Materials and Methods

### 2.1. Experimental Setup and Uncertainty Quantification for 1 g of CH_4_ h^−1^ Emission

Controlled release experiments were conducted at Colorado State University’s Methane Emission Technology Evaluation Center (METEC) in Fort Collins, CO, USA to quantify the uncertainty of each method for 1 g CH_4_ h^−1^ emission. In each test, an emission amount of 1 g CH_4_ h^−1^ was established using an MC-500SCCM-D-PCV03/5M mass flow controller (Alicat Scientific, Inc., Tucson, AZ, USA) and 100% CH_4_ gas supplied via a compressed gas cylinder, emitted through 1 m of PTFE tubing. The dynamic chambers, an HF sampler, and a GP approach were used to quantify the emission in triplicate. The uncertainty was then calculated as the percentage difference between the average quantified emission and known emission rate (1 g CH_4_ h^−1^). Results are presented as the average accuracy of the three experiments (*A_r_*; %) and the upper (*U_u_*; %) and lower (*U_l_*; %) uncertainty bounds of the three individual experiments.

### 2.2. Dynamic Chamber

Following the guidelines defined by the ACR [7], the dynamic chamber comprised a rigid container (0.12 m^3^) enclosing the source, with an inlet and outlet flow of air through the chamber, and a propeller used to mix the gas and the air inside the chamber. As such, the ACR-prescribed chamber can be heavy, difficult to transport, and less able to fit over large well heads. The CH_4_ flux (*Q*, g s^−1^) was calculated (Equation (1)) from the CH_4_ concentration at steady state (*X_ss_*, g m^−3^), the background CH_4_ concentration (*X_b_*, g m^−3^), and the flow of air through the chamber (*F*, m^3^ s^−1^) [4].

(1) $$Q=F\cdot \left(X_{ss}-X_{b}\right)$$

**Figure 1:**
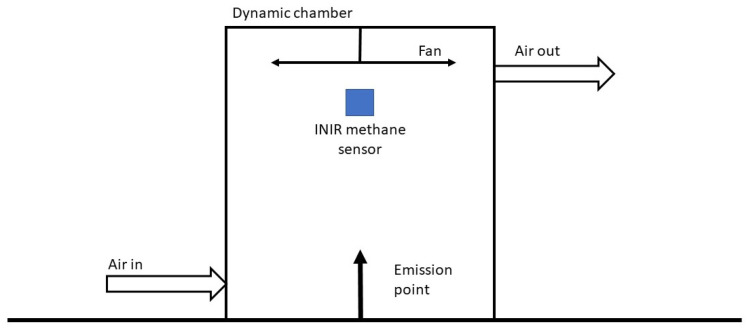
Schematic of the dynamic/mobile flux chambers.

A single chamber with a volume of 0.12 m^3^ (Figure 1), open at one end, was placed over the 1 g CH_4_ h^−1^ emission, and air was passed through the chamber at a constant rate of 8.5 L per minute (L min^−1^). Air flow rate was measured using a Cole-Palmer flowmeter. Methane concentrations inside the chamber were measured using an INIR-ME100% sensor (SGX Europe, Katowice, Poland) mounted inside the chamber. The INIR sensor occupies a relatively small volume (5 cm^3^) and was fixed near to the center of the chamber. This was below the fan and above the emission source with the aim of measuring the methane concentration in the volume of air that is most mixed by the fan. The INIR-ME100% is an integrated infrared sensor that, once calibrated, can measure the volumetric CH_4_ mixing ratios between 300 ppm and 100% methane to an accuracy of ±5%. The sensor has a T90 response time of 20 s. Data were transmitted from the INIR-ME100% to an Arduino Uno and stored on an SD card every 5 s. Technical details of the INIR integration with the Arduino and the code used to log the Arduino data are presented in Supplementary Materials Sections S1 and S2, respectively. Gas was turned on, and the pump was started after 3 min. The *X_ss_* was taken as the average of the five-minute CH_4_ concentration once it had plateaued, and the experiment was repeated three times. 

### 2.3. Mobile Chamber

As most abandoned wells are remote from roads, and many are large pieces of equipment [4,11], quantifying emissions using a rigid chamber, as prescribed by the ACR, is challenging as the chamber must be large enough to enclose the entire well head or pumpjack. For very large well heads or pumpjacks, the task of transporting a rigid chamber to the well head would be impractical. More suited to the task is something lightweight that can be dismantled, carried across rough terrain, and then assembled around the emission source.

To address the mobility issue, a mobile alternative to the dynamic chamber specified by the ACR was developed and tested. Instead of a rigid plastic container, plastic poles were used to form a 0.12 m^3^ rectangular cuboid, and polyethylene sheeting was used to form the sides of the chamber (Figure S5). A bespoke chamber can be constructed at each measurement site in five minutes to fit any well head/pumpjack; therefore, emissions from unusually shaped sources can be quantified. All other parts of the dynamic chamber (fan, inlet, outlet, and INIR sensor) and the quantification method described for the dynamic chamber in Section 2.2 remained the same apart from the pump, which drew air through the chamber at 6.0 L min^−1^. Unlike the dynamic chamber method, the gas was started and then the pump started. The method for the mobile chamber was changed from the rigid dynamic chamber’s method to accommodate practical issues and to ensure that the tubing carrying the gas into the chamber could be held in place while turning on the gas.

### 2.4. Semtech Hi-Flow

Both the dynamic and mobile chambers are homemade and require time and effort to optimize the system. One off-the-shelf alternative is the Semtech Hi-Flow 2 sampler (Heath Consultants Inc., Houston, TX, USA, www.heathus.com, accessed on 14 November 2023). An HF sampler draws air into a measurement chamber at a known rate, and the concentration of CH_4_ in the air is measured using tunable diode laser absorption spectroscopy, and the emission rate is calculated (Equation (1)). The Semtech HF draws air at a rate between 140 and 850 L min^−1^ and can measure CH_4_ emissions between 0.6 g CH_4_ h^−1^ and 28 kg CH_4_ h^−1^ to a stated accuracy of ±20% [25]. No other data are required to calculate an emission. The bag containing the hose end of the HF sampler was placed over the 1 g CH_4_ h^−1^ point source, and the instrument was turned on with the flow rate of 495 L min^−1^. Measurements were repeated three times, and the average emission was calculated. The HF sampler used in this study was calibrated monthly, as recommended by the manufacturer.

### 2.5. Gaussian Plume (GP) Approach

A Gaussian plume approach was used to calculate the average emission downwind of the source of the gas. The emission rate (*Q*, g s^−1^) can be calculated from the methane concentration directly downwind of the source (*Χ*, g m^−3^), the average wind speed (*u*, m s^−1^), and the standard deviation of the lateral (*σ_y_*, m) and vertical (*σ_z_*, m) mixing ratio distributions (Equation (2)). The *σ_y_* and *σ_z_* were calculated from the Pasquill–Gifford stability class of air (US EPA, 1995). The GP model assumes that both the emission rates and wind speed are constant.

(2) $$Q=2\pi u\sigma_{y}\sigma_{z}X$$

Methane concentrations were measured for 5 min 0.5 m directly downwind of the emission point using an ABB GLA131-GGA Micro portable Greenhouse Gas Analyzer (MGGA; ABB Inc., Quebec, CA, USA). ABB states that the MGGA has a measurement precision of <2 ppb (1σ at 1 Hz) over an operating range of 0.1 to 100 ppm. Wind speed and air temperature data were collected using a Kestrel 5500 weather meter collocated with the analyzer inlet. The Kestrel 5500 measures wind speed from 0.6 to 40.0 m s^−1^ (resolution 0.1 m s^−1^, accuracy ±3%), air temperature between −29 °C and 55 °C (resolution 0.1 °C, accuracy ±0.5%), atmospheric pressure from 750 mbar to 1100 mbar (resolution 0.1 mbar, accuracy ±1.5 mbar), and relative humidity between 5 and 95% (resolution 0.1%, accuracy ±3% RH).

## 3. Results

### 3.1. Dynamic Chamber

**Figure 2:**
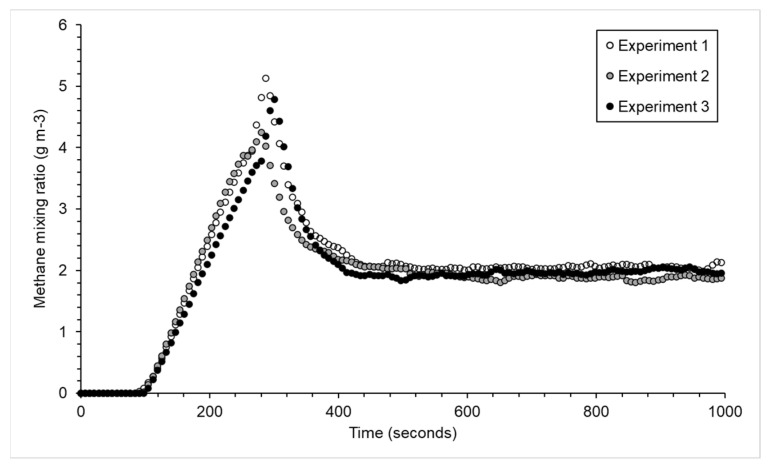
Methane concentration change inside a chamber with a volume of 0.12 m^3^ placed over an emission of 1 g CH_4_ h^−1^. After 3 min, a pump of 8.5 L min^−1^ was turned on.

Once the chamber was placed over the emission point, the concentration inside the chamber increased from 0.023 g m^−3^ CH_4_ s^−1^ (~35 ppmv CH_4_ s^−1^) to around 4.5 g m^−3^ (~7000 ppmv). The pump was turned on after 3 min, and the concentration became relatively constant after another 3 min (Figure 2). The *X_ss_* inside the chamber for the three experiments with a flow rate of 8.5 L min^−1^ was 1.99 ± 0.1 g m^−3^, which corresponds to an average emission rate of 1.01 ± 0.06 g CH_4_ h^−1^ (Table 1). 

One point of note is that the rate of change of the CH_4_ inside the chamber can be used to calculate the emission, as is carried out using a static chamber approach [26]. The average emission calculated from the linear increase in the methane concentration inside the chamber of 0.023 g m^−3^ CH_4_ s^−1^, the diameter of the chamber of 0.25 cm, and the height of chamber of 0.40 m would be 1.64 g CH_4_ h^−1^ with an overestimate of +64% (range +51% to +79%). Additionally, a chamber of 0.12 m^3^ placed over an emission at a rate of 1 g CH_4_ h^−1^ would become explosive after 23 min.

### 3.2. Mobile Chamber

**Figure 3:**
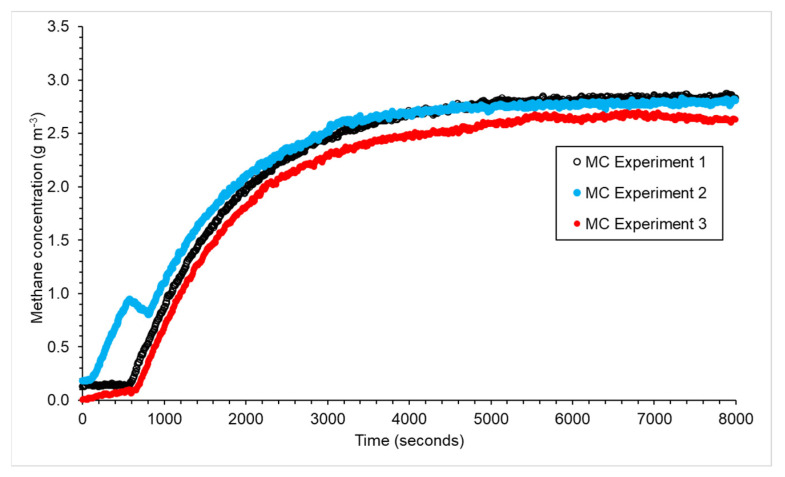
Methane concentration change inside a chamber with a volume of 0.12 m^3^ with a flow of air of 6 L min^−1^ placed over an emission of 1 g CH_4_ h^−1^.

The method used for the dynamic chamber was adapted for the mobile chamber as the inlet needed to be held in place when the gas was turned on. This was only a shortcoming that was specific to our instrumental setup and is not a characteristic shortcoming of the mobile chamber methodology. Therefore, it took longer for *X_ss_* to be reached; it took 1 h 20 min instead of 6 min (Figure 3). The *X_ss_* inside the chamber with a volume of 0.12 m^3^ for the three experiments with a flow rate of 6 L min^−1^ was 2.76 ± 0.08 g m^−3^, which corresponds to an average emission rate of 0.99 ± 0.04 g CH_4_ h^−1^ (Table 1).

### 3.3. Hi-Flow Sampler

Three tests were conducted with the Hi-Flow sampler. Flow rates of 247.5, 346.5, and 495 L min^−1^ were used, which corresponded to the calculated average emission rates of 1.02 ± 0.06 g CH_4_ h^−1^, 1.09 ± 0.04 g CH_4_ h^−1^, and 1.18 ± 0.05 g CH_4_ h^−1^, respectively (Table 1). This suggests that even though the Hi-Flow sampler maintains precision, as the flow rate of the instrument increases, the accuracy decreases. The differences between the average calculated emission and the known emission of 1 g CH_4_ h^−1^ were +2.2%, +9.1%, and +17.8% for the 247.5, 346.5, and 495.0 L min^−1^ flow rates, respectively.

### 3.4. Gaussian Plume Approach

The methane concentrations and wind speed were measured 50 cm away from the emission point for 5 min and then averaged. As the room was temperature-controlled and the ground had the same temperature as the air, it was judged that there was little vertical motion of the air, but the wind speed was not enough (<2 m s^−1^) to consider the air neutral. As a result, the Pasquill–Gifford stability class was assigned class C. Over the three experiments, the downwind methane mixing ratios averaged 15.7 ppm (Table 2) and corresponded to an average calculated emission rate of 0.97 ± 0.07 g CH_4_ h^−1^.

### 3.5. Overall Results

Our results show that the average emission from the three measurements made using the dynamic chamber (*A_r_* = +1%), mobile chamber (*A_r_* = −2%), the GP approach (*A_r_* = −3%), and the HF (*A_r_* = +3%) were all able to quantify the emission from an emission source of 1 g of CH_4_ h^−1^ after triplicate experiments to within the uncertainty required by the FWOP guidelines (±30%) and ACR (±20%) criteria (Table 3). The HF and GP approach were the least precise of these methods, while the ACR-prescribed dynamic chamber was the most precise. 

## 4. Discussion

Using triplicate measurements, all of the methods described above can be used to quantify emissions of 1 g CH_4_ h^−1^ to within ±3%, with all individual emission measurements falling to ±10% of the 1 g CH_4_ h^−1^ threshold defined by the BLM. This suggests that any of these methods presented here can be used to satisfy both the FWOP guidelines (±30%) and the ACR (±20%) criteria. The GP approach has been shown in the study to work when the detector is directly downwind of the source, i.e., y = 0 m, and care should be taken to ensure that this remains true, as any lateral displacement greatly affects the uncertainty as it is not parameterized for small distances [24]. The Semtech Hi-Flow 2 can quantify emissions to +8/−5% and has the added feature of directly calculating and displaying emissions after the measurement. 

The results suggest that, despite the design and construction criteria defined by the ACR, CH_4_ emissions of 1 g CH_4_ h^−1^ can be quantified using any suitably designed dynamic chamber to within ±6% irrespective of the material, number of parts, and input hose length. The specifications of the material, shape, and number of chamber parts do not make a discernible difference to the quantification uncertainty and suggest that the flow of air through the chamber could negate the expected effects of adsorption, temperature increase, and circulation. This means that a low-cost and collapsible chamber can be used to quickly quantify emissions from abandoned wells in remote areas.

Given that all methods presented here fall within the uncertainty bound required by both the FOWP and ACR, practical considerations may limit the measurement method choice. For a well-resourced operation aiming to quantify many abandoned wells quickly, the HF may be the most attractive option as it directly outputs an emission rate in g h^−1^. The key shortcoming of the HF method is being confident that all the emitted gas goes into the HF sampler. This is sometimes a real challenge in the field with emissions coming from awkward parts of the Christmas tree configuration or the pumpjack, but it can be overcome by using an optical gas imaging (OGI) camera in tandem to ensure that all the gas is collected. However, OGI cameras are expensive (>$50k), and they may not be able to detect emissions of less than 20 g of CH_4_ h^−1^ [19,20].

The GP approach overcomes “bagging the leak” by making a downwind measurement of the CH_4_ concentration and coupling it with the meteorological data in a relatively simple algorithm (Equation (2)). One shortcoming of the GP approach is that it requires the use of a trace CH_4_ analyzer, i.e., an instrument that can quantify concentrations of parts per billion. Even though there are several instruments on the market, the costs are high (between USD 30k and USD 50k), and the instrument typically requires an experienced operator. All of this means that a measurement operation is unlikely to have many measurement units running at one time.

Even though they require a longer setup time, dynamic chambers are a lower-cost alternative to HF and GP approaches, which require instrumentation costing in excess of USD 20,000. This study shows that the frame of a mobile dynamic chamber costing around USD 300 to construct can be built around an emission point and sealed with a material to quantify emissions to the uncertainty required by the FOWP and ACR. Chambers were first used to measure the CH_4_ emission from abandoned wells in Appalachia [4,26] as they were inexpensive, and CH_4_ concentrations could be collected as gas samples in a glass vial and assayed at a later date using a gas chromatograph. The shortcomings of this approach were that the chamber was typically difficult to transport. The chamber must enclose the entire source, which were sometimes meters tall and wide, and an analysis using the gas chromatograph was time consuming. The results from this study have demonstrated that a dynamic chamber built around the emission point, regardless of the shape and construction material, can be used to quantify emissions as low as 1 g of CH_4_ h^−1^ to ±6%. It is possible that smaller, more easily transportable chambers could be used; however, methane may not completely mixed in a smaller volume chamber. Regardless, the chamber used in this study (volume of 0.12 m^3^) readily fits inside a backpack and is easily transported.

The results also indicate that a relatively low-cost CH_4_ sensor, the SGX INIR-ME100% interfaced to a low-cost, low-power logger (the full details of which can be found in the Supplementary Information), can be used to quantify the volumetric CH_4_ mixing ratios in real time, indicating that the emission rates can be calculated after a 5 min measurement using a simple algorithm (Equation (1)).

In conclusion, we suggest that well-resourced operations could use the GP and HF methods to quantify emissions, but this comes with the significant expense of buying the instrumentation. A much lower cost approach is the mobile dynamic chamber, which can be operated by technicians with minimal training to measure the CH_4_ emissions from abandoned oil and gas wells in remote locations. As the cost of the chamber is low, many chambers can be deployed simultaneously, making quantification from thousands of abandoned wells a realistic possibility. 

## Figures and Tables

**Table 1:** Collated data from the dynamic chamber (DC), mobile chamber (MC), and Hi-Flow (HF) sampler being used to quantify the emissions from a source of 1 g CH_4_ h^−1^. “Expt.” refers to the individual experiment number, F is the flow rate passing through the chamber/Hi-Flow, X_ss_ is the steady-state concentration inside the chamber, Q is the calculated emission rate, and U is the uncertainty in the calculated emission when compared to the known emission rate of 1 g CH_4_ h^−1^.

Method	Expt.	*F* (L min^−1^)	*X_ss_* (g m^−3^)	*Q* (g h^−1^)	*U* (%)
DC	1	8.5	2.07	1.05	5.35
DC	2	8.5	1.88	0.96	−4.02
DC	3	8.5	1.99	1.01	1.31
MC	1	6.0	2.84	1.02	2.13
MC	2	6.0	2.79	1.00	0.43
MC	3	6.0	2.66	0.96	−4.40
HF Rate 1	1	247.5	0.073	1.08	8.00
HF Rate 1	2	247.5	0.065	0.96	−4.20
HF Rate 1	3	247.5	0.069	1.03	2.80
HF Rate 2	4	346.5	0.054	1.11	11.30
HF Rate 2	5	346.5	0.053	1.11	11.20
HF Rate 2	6	346.5	0.050	1.05	4.80
HF Rate 3	7	495	0.039	1.16	15.50
HF Rate 3	8	495	0.039	1.15	15.20
HF Rate 3	9	495	0.041	1.23	22.70

**Table 2:** Downwind measurements: wind speed (WS), background CH4 mixing ratio (BMR), CH_4_ mixing ration 0.5 m downwind (DMR), the Pasquill–Gifford stability class (PGSC), the distance between the source and detector inlet (d, cm), the height of the source and inlet (h, cm), the emission calculated using the Gaussian Plume approach (QGP), and the percentage uncertainty of the Gaussian Plume estimate (UGP).

WS (m/s)	*B_MR_* (ppm)	*D_MR_* (ppm)	*PGSC*	*d* (cm)	*h* (cm)	*Q_GP_* (g/h)	*U_GP_* (%)
1.15	2.29	16.56	C	50	0.5	0.99	−1.2
1.26	2.19	13.68	C	50	0.5	0.91	−8.8
1.10	2.20	16.94	C	50	0.5	1.03	+2.1

**Table 3:** Collated results for the dynamic and mobile chambers, Gaussian Plume (GP) approach, and Hi-Flow (HF). Results show the average emission rates (Qav; g h^−1^) after triplicate experiments, the average bias (A_r_; %), and the upper (U_u_; %) and lowest (U_l_; %) uncertainty bounds of the three experiments.

Method	*Q_av_* (g h^−1^)	*A_r_* (%)	*U_u_* (%)	*U_l_* (%)
Dynamic chamber	1.01	+0.9	+5.4	−4.0
Mobile chamber	0.99	−1.4	+1.7	−5.8
GP	0.97	−2.6	+2.1	−8.8
HF	1.02	+2.2	+8.0	−4.2

## Data Availability

All data for this study are found in the Supplementary Materials.

## Supplementary Materials

The following supporting information can be downloaded at https://www.mdpi.com/article/10.3390/s23229246/s1. Figure S1. Description and data format details given in the INIR manual. Figure S2. Sample INIR output data. Figure S3. Sc hematic of level shifter required to convert the 3.3V INIR data output from 3.3 Volts to 5 Volts. Figure S4. Schematic of INIR Arduino Logger with LCD display. Figure S5. The mobile dynamic chamber. Plastic poles were used to form a 0.12 m^3^ rectangular cuboid, and polyethylene sheeting (Saran wrap) was used to form the sides of the chamber and enclose the leak.
