# Supplementary material for: Uncertainty Quantification of Methods Used to Measure Methane Emissions of 1 g CH4 h−1
Source: Sensors (Basel). 2023 Nov 17;23(22):9246. doi: 10.3390/s23229246 (PMC10675349; doi:10.3390/s23229246)
Supplement: Supplementary file 1 [file sensors-23-09246-s001.zip › sensors-2699685-SI.pdf]

sensors

MDPI

Article

Uncertainty Quantification of Methods Used to Measure Methane Emissions of 1 g CH_4 h^{-1}

Stuart N. Riddick [1,*], Mercy Mbua [1], John C. Riddick [2], Cade Houlihan [1], Anna Hodshire [1] and Daniel J. Zimmerle [1]

1 Energy Institute, Colorado State University, Fort Collins, CO 80524, USA; mercy.mbua@colostate.edu (M.M.); cade.houlihan@colostate.edu (C.H.); anna.hodshire@colostate.edu (A.H.); dan.zimmerle@colostate.edu (D.J.Z.)
2 Independent Researcher, Lockerbie, Scotland; john_riddick@hotmail.com
* Correspondence: stuart.riddick@colostate.edu

Supplementary Materials

1. INIR Datalogger Construction

1.1. Introduction

The following are development and construction notes detailing the interface of an INIR methane sensor to an Arduino Uno microcontroller and data logger.

Two versions were built:

1 INIR sensor, Liquid Crystal Data Display, data logged to SD card;

2 INIR sensor, data displayed through Bluetooth to a phone, data logged to SD card.

The methane concentrations are output from the INIR sensor as a serial binary data stream; these data are interfaced into an Arduino, decoded, and stored along with the time and date to a file on an SD card.

1.2. INIR Output

This sensor is run in a continuous mode with the gas concentrations being output once per second at a serial data stream of 38400 baud, with eight data bits, one stop bit, no parity, and no handshake. The description and data format details given in the INIR manual are ambiguous (Figure S1).

```
[                       // Start Character 0x0000005B (HEX)
0xAAAAAAAA                 // Gas concentration in PPM (HEX)
0xAAAAAAAA                 // Faults (HEX)
0xAAAAAAAA                 // Sensor Temperature (HEX)
0xAAAAAAAA                 // CRC
0xAAAAAAAA                 // 1's Compliment of CRC
 ]                      // End Character 0x0000005Du (HEX)
```

Figure S1. Description and data format details given in the INIR manual.

The first problem was the terminators of each data sample. In the manual, they are shown as [and], which the manual states, when decoded from ASCII to their hexadecimal equivalent, are 0 × 5b and 0 × 5d. But when the data are analyzed by recording the output string using a terminal emulator on a PC, the INIR output is as shown in Figure S2.

```
0x000: 3030 3030 3030 3562 0D0A 3030 3030 3030  000005b..000000
0x010: 3030 0D0A 6131 6161 6161 3161 0D0A 3030  00..a1aaaa1a..00
0x020: 3030 3062 3631 0D0A 3030 3030 3032 6436  000b61..000002d6
0x030: 0D0A 6666 6666 6664 3239 0D0A 3030 3030  ..fffffd29..0000
0x040: 3030 3564 0D0A 3030 3030 3030 3562 0D0A  005d..0000005b..
```

Figure S2. Sample INIR output data.

From the above output, the " [" character referred to in the manual is actually 2 bytes, 0x35 for an ASCII of "5" and 0 × 62 for an ASCII of "b"; the same is true for the terminator character "]", 0 × 35 and 0 × 62. Secondly, each line of data is terminated via carriage return and line feed (0 × 0D, 0 × 0A) characters, which is not noted in the manual. Finally, the output level of the digital RS232 data string is equivalent to the TTL and not RS232. This means that the digital levels are reversed, i.e., a logic 1 in TTL is a logic 0 in the RS232 format. The function of the two CD4049 invertors shown in the circuit is to ensure that the signal levels are correct. They may be omitted in the final circuit, as tests have shown that the output of the 3.3V to 5 V level shifter can be input directly into the Arduino. This inversion did not cause problems, as this is the level in which the Arduino serial input is decoded.

1.3. Arduino Interface

Methane concentrations are detected via the INIR, which digitizes the sample and outputs the data as a serial stream, with 38400 baud, eight data bits, no parity, and one stop bit at a 3.3 Voltage level. The Arduino input levels are between 5 Volts and 0 Volts, and in order for the Arduino to decode the INIR data, it is level-shifted from 3.3 Volts to 5 Volts (Figures S1–S3).

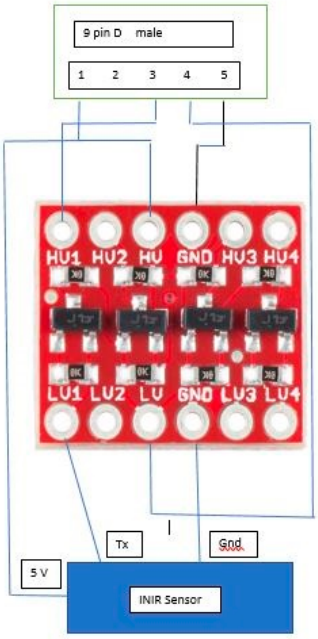

Figure S3. Schematic of level shifter required to convert the 3.3V INIR data output from 3.3 Volts to 5 Volts.

Arduino to INIR Interface 9 pin "D" male

1. 5 Volts;
2.
3. INIR RS232;

4. 3.3 Volts;
5. Ground.

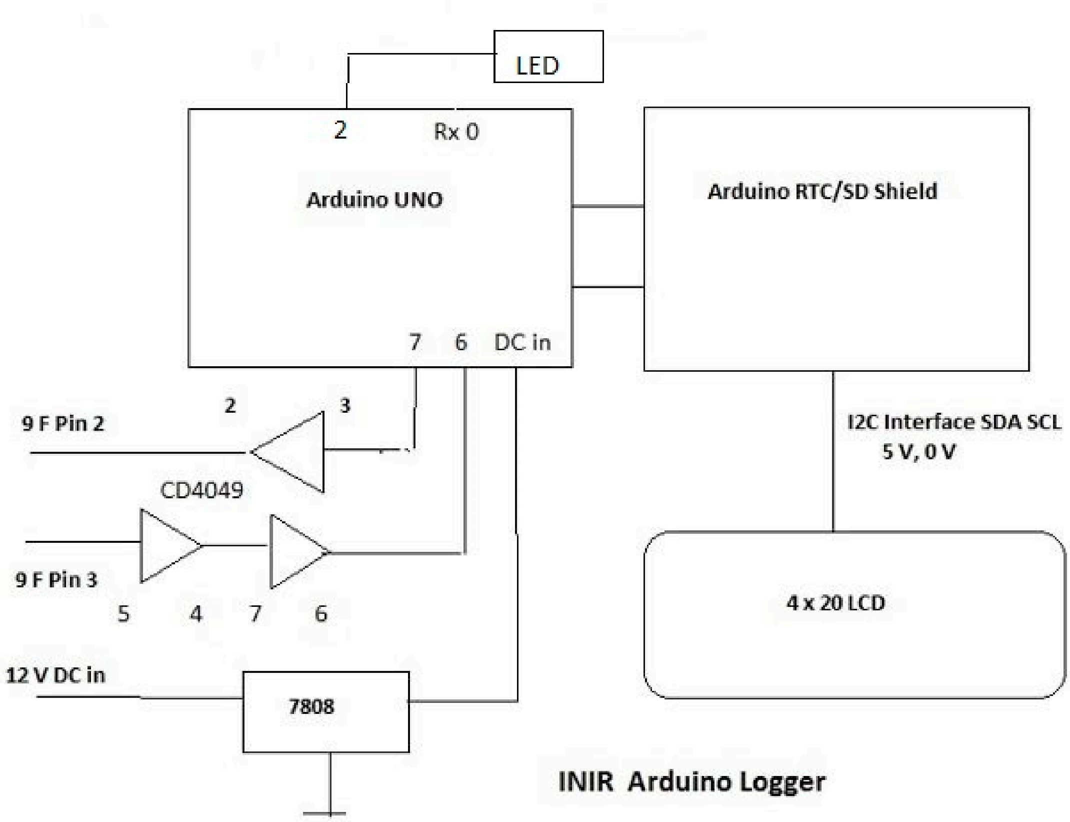

Figure S4. Schematic of INIR Arduino Logger with LCD display.

1.4. Power Supply

The Arduino, INIR, and all associated hardware operate from a single 5 Volt source. But for ease of operation, both versions of this data logger can be powered from a single 12 Volt battery using an LM7808 regulator to reduce the battery Voltage to 8 Volts; this powers the Arduino and all of the hardware through the Uno 2.1 mm jack socket. An amount of 12 Volts DC are input to LM7808, which regulates 12–14 Volts to 8 Volts by supplying the Arduino 2.1 mm jack plug; the center pin has +8 Volts, and the outer pin has 0 Volts.

1.4.1. DC Power Requirements

Liquid Crystal Display (LCD) version 150 mA;
Liquid Crystal Display version with LCD switched off 102 mA;
Bluetooth version 130 mA.

The above figure is used to calculate the operating times from a 12 Volt battery as follows:

LCD (display), approximately 6 hours/1AH or 4AH/day;
Bluetooth version, 8 hours/1AH or 3 AH/day.

To allow for battery inefficiencies, temperature, etc., a 40AH automobile battery will power a logger for 1 week.

1.5. Arduino Software

A program has been written for the data display on an LCD screen. The complete Arduino script is presented in the Supplementary Material, Section 2.

1.6. Arduino Construction

Parts list:

Plastic case, the prototype box measuring 24 × 16 × 9 cm, but a smaller case would suit;

Arduino Uno;

Arduino SD and RTC shield, RTC Battery;

4 × 20 Liquid Cristal Display (LCD) supporting I2C interface;

CD4049 cmos integrated circuit, 16 pin wire wrap DIL socket;

LM 7808 DC/DC regulator;

Regulator heat sink for LM7808;

Arduino DC connector 2.2 mm (see diagram above);

Red and Black 4 mm sockets for DC input;

Terminal strip to connect Arduino shield outputs to 9-way D connectors;

Stand-off supports to mount Arduino in box;

9-way D male socket;

9-way D female socket;

5mm LED, 560 ohm resistor;

Depending on whether the user uses an iPhone or Android, a compatible Arduino Bluetooth module is needed, either a BT05 or Android-compatible device;

Sparkfun 3.3 V to 5 Volt level shifter—bidirectional https://www.sparkfun.com/products/12009;

1Gb SD card;

Suitable case for INIR and 3.3V to 5 Volt level shifter.

1.7. Input/Output Wiring

Nine-way D male Wired to TX0/TX0 Arduino Hardware Serial port:

1. + 5 Volts;
2. Not used;
3. Serial data input to CD4049 pin 5;
4. 3.3 Volts;
5. Ground.

2. INIR Arduino Logging Code

```
// Read_Port_SD2_Decode_Time_LCD_MON
// Program decodes INIR sample and writes it to
// SD card
// 99 ID is written along with date and time
// Logger operation is displayed on 4x20 LCD
#include <stdlib.h>
#include <SoftwareSerial.h>
#include <SPI.h>
#include <SD.h>
#include <Wire.h>
#include "RTClib.h"
#include <LiquidCrystal_I2C.h>
LiquidCrystal_I2C lcd(0 × 27, 20, 4);
```

```
SoftwareSerial MONSerial(6,7); // RX, TX   Monitor
byte ID[3];
byte datin[20];
int led = 2;
int count_in = 0;
const int chipSelect = 10;
float Result;
int yy;
int Y20;
int mm;
int dd;
int h;
int m;
int s;

const int DS1307 = 0x68; // Address of DS1307
RTC_DS1307 rtc;
char filename[] = "00000000.txt";
void setup(){
    Serial.begin(9600);
    pinMode(led, OUTPUT);   // LED
    MONSerial.begin(38400);

    Wire.begin();
    lcd.init();
    lcd.backlight();
    lcd.print("INIR Monitor   V1");
    delay(1000);

    if (!SD.begin(chipSelect)) {
     lcd.clear();
     lcd.setCursor(0, 0);
     lcd.print("SD Not Recognised ");
    while (1);
  }
  if (! rtc.begin()) {
     lcd.setCursor(0, 1);
     lcd.print("RTC Not Recognised ");
    while (1);
  }

  //Serial.println("Init");
```

```
  lcd.clear();
  lcd.setCursor(0, 0);
  lcd.print("RTC SETUP ");
  lcd.setCursor(0, 1);
  lcd.print("SD SETUP ");
  lcd.setCursor(0, 3);
  lcd.print("Collecting Data");
  delay(1000);

}
void loop(){
   count_in = 12;
   //    Get INIR data
        MONSerial.flush();
        MONSerial.listen();

         count_in = MONSerial.find("0000005b/n/r", 10);
         count_in = MONSerial.readBytesUntil("0000005d/n/r", datin, 62);
     if( datin[10] == 0 × 30){
     digitalWrite(led, HIGH);
     delay(100);
     digitalWrite(led, LOW);
    // SMP_LED(); // Flash LED Data Rx
    if(count_in > 11){
   String dataString = "";   // Clear Output string
   dataString += '9';   // Identify Sensor
   dataString += '9';
   dataString += " ";
    //   Read the time
  DateTime now =rtc.now();
  yy = now.year();
  Y20 = yy-2000;
   dataString += yy;
   dataString += " ";
   mm=now.month();
   dataString += mm;
   dataString += " ";
   dd = now.day();
   dataString += dd;
   dataString += " ";
   h = now.hour();
   dataString += h;
```

```
   dataString += " ";
   m = now.minute();
   dataString += m;
   dataString += " ";
   s = now.second();
   dataString += s;
   dataString += " ";

    Decode_INIR();
    dataString += Result;
    // Debug code
    /*  BlueSerial.println(datin[12],HEX);
        BlueSerial.print(datin[13],HEX);
        BlueSerial.print(datin[14],HEX);
        BlueSerial.print(datin[15],HEX);
        BlueSerial.print(datin[16],HEX);
        BlueSerial.println(datin[17],HEX);
     */

// Write data to IIyymmdd.txt
   getFilename(filename);
   File dataFile = SD.open(filename, FILE_WRITE);

  // if the file is available, write to it:
       if (dataFile) {
        dataFile.println(dataString);
        dataFile.close();
         lcdout_1(); // Send result to LCD
             }
           }
       }
       delay(100);
}


void Decode_INIR()
{
unsigned int R1;
unsigned int R2;
unsigned int R3;
unsigned int R4;
unsigned int R5;
```

```
unsigned int R6;
  R1 = datin[17] & 0 × 0F;
  R2 = (datin[16] & 0 × 0F);
  R3 = datin[15] & 0 × 0F;
  R4 = (datin[14] & 0 × 0F);
  R5 = datin[13] & 0 × 0F;
  R6 = (datin[12] & 0 × 0F);
  Result = (R5*65536)+(R4*4096)+(R3*256)+(R2*16)+R1;
}

void getFilename(char * filename) {
  int nyy;
   filename[0] = '9';
   filename[1] = '9';
   nyy = yy - 2000;
   filename[2] = (nyy)/10 + '0';
   filename[3] = nyy%10 + '0';
   filename[4] = mm/10 + '0';
   filename[5] = mm%10 + '0';
   filename[6] = dd/10 + '0';
   filename[7] = dd%10 + '0';

   filename[8] = '.';
   filename[9] = 'T';
   filename[10] = 'X';
   filename[11] = 'T';
    return;
  }
   // Display Date, Time and Data on LCD
void lcdout_1()
{
   lcd.clear();
   // Print line 1
  lcd.setCursor(0, 0);
  //lcd.print("Sensor 99 ");
  //lcd.print(" ");
  lcd.print(String(Y20));
  //lcd.print(" ");
   if (mm <10){(lcd.print('0'));}
  lcd.print(String(mm));
  //lcd.print(" ");
   if (dd <10){(lcd.print('0'));}
```

```
  lcd.print(String(dd));
  lcd.print(" ");
  if (h <10){(lcd.print('0'));}
  lcd.print(String(h));
  //lcd.print(":");
  if (m <10){(lcd.print('0'));}
  lcd.print(String(m));
  //lcd.print(":");
  if (s <10){(lcd.print('0'));}
  lcd.print(String(s));
   // Print line 2
  lcd.setCursor(0, 1);
  lcd.print("Gas ");
  lcd.print(Result);
  lcd.setCursor(0, 2);
  lcd.print("Bytes in ");
  lcd.print(count_in);
// lcd.print(" ");// Debug Gas in Data
// lcd.print(datin[16]);
// lcd.print(datin[17]);
  lcd.setCursor(0, 3);
  lcd.print("Writing Data to SD");

}
```

3. Mobile Dynamic Chamber

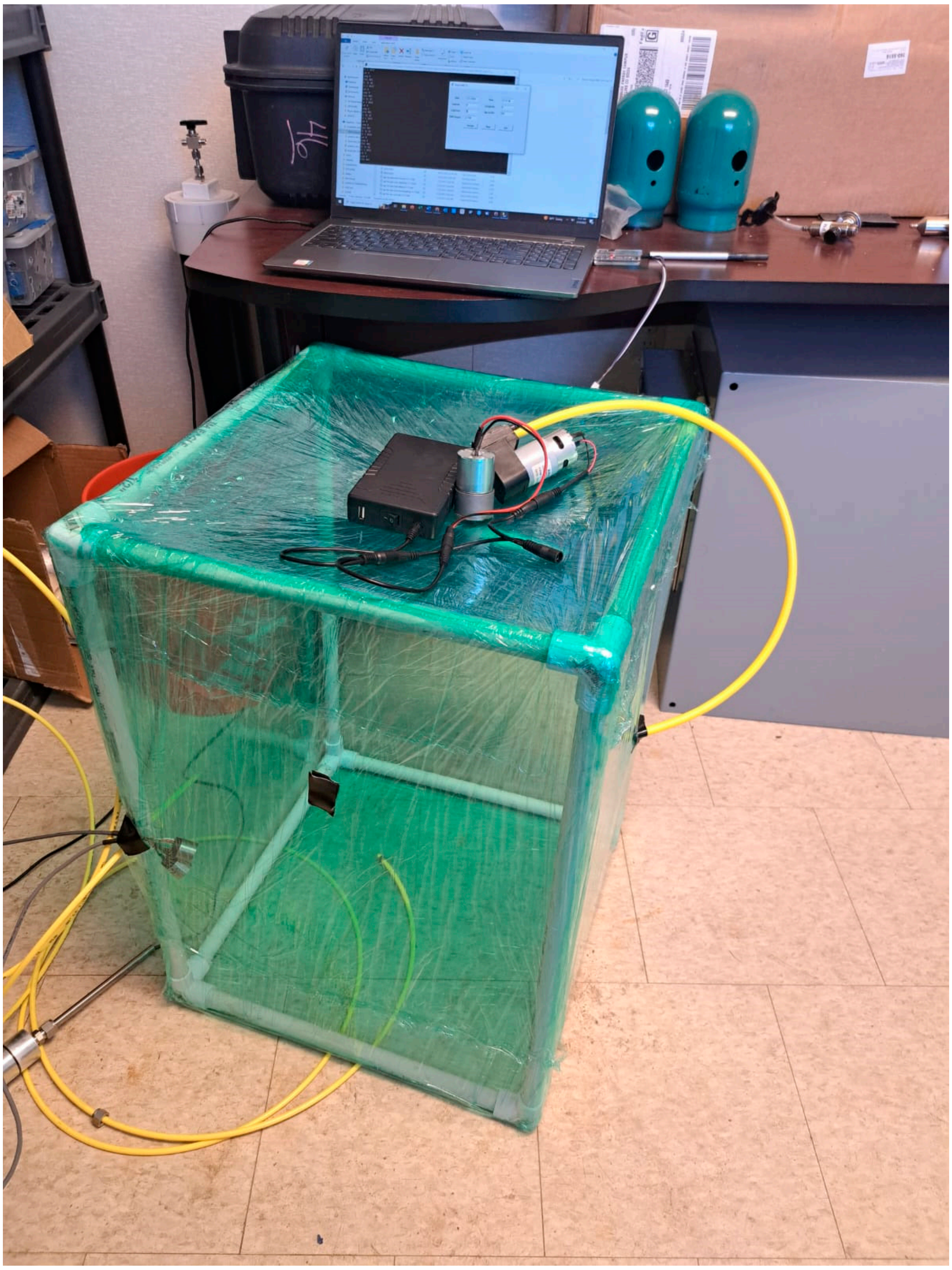

Figure S5. The mobile dynamic chamber. Plastic poles were used to form a 0.12 m^3 rectangular cuboid, and polyethylene sheeting (Saran wrap) was used to form the sides of the chamber and enclose the leak.
